# Sexual and reproductive health needs and practices of female sex workers in Papua New Guinea: findings from a biobehavioral survey *Kauntim mi tu* (‘Count me too’)

**DOI:** 10.1186/s13690-022-00926-y

**Published:** 2022-09-05

**Authors:** Damian Weikum, Angela Kelly-Hanku, Ruthy Neo-Boli, Herick Aeno, Steven G. Badman, Lisa M. Vallely, Barne Willie, Martha Kupul, Parker Hou, Angelyn Amos, Rebecca Narokobi, Simon Pekon, Kelsey Coy, Johanna Wapling, Janet Gare, John M. Kaldor, Andrew J. Vallely, Avi J. Hakim, on behalf of the Kauntim mi tu Study Team

**Affiliations:** 1 US Centers for Disease Control and Prevention, Atlanta, USA; 2 Public Health Institute, Oakland, USA; 3 Sexual and Reproductive Health Unit, Papua New Guinea Institute of Medical Research, Goroka, Eastern Highlands Province Papua New Guinea; 4 Public Health Intervention Research Program, Kirby Institute for Infection and Immunity in Society, UNSW Sydney, Sydney, NSW Australia

**Keywords:** Papua New Guinea, Female sex workers, Sexual and reproductive health, HIV, Antenatal care, Moderately or highly effective contraception

## Abstract

**Background:**

Little research has explored the sexual and reproductive health (SRH) experience of female sex workers (FSW), including girls aged < 18 years who are commercially sexually exploited (CSE), in Papua New Guinea (PNG). This paper describes the SRH history of FSW and CSE girls and factors associated with their use of moderately or highly effective contraceptive methods in three settings in PNG.

**Methods:**

From 2016 to 2017, respondent-driven sampling (RDS) surveys were conducted among FSW and CSE girls in Port Moresby, Lae, and Mt. Hagen. FSW and CSE girls who were born female, aged ≥12 years, sold or exchanged vaginal sex in the past 6 months, spoke English or Tok Pisin, and had a valid RDS study coupon were eligible to participate. Interviews were conducted face-to-face and participants were offered rapid routine HIV and syphilis testing. Survey logistic regression procedures were used to identify factors associated with the use of moderately or highly effective contraceptive methods. Weighted data analysis was conducted.

**Results:**

A total of 2901 FSW and CSE girls (Port Moresby, 673; Lae, 709; and Mt. Hagen, 709) were enrolled. The proportion using moderately or highly effective contraceptive methods was 37.7% in Port Moresby, 30.9% in Lae, and 26.5% in Mt. Hagen. After adjusting for covariates, factors significantly associated with the use of moderately or highly effective contraceptive methods in Port Moresby were being age 20–24, being married, being divorced or separated, having one or more dependent children, being away from home for more than 1 month in the last 6 months, and having tested HIV negative. No factors were significantly associated in Lae or Mt. Hagen. ANC attendance amongst FSW and CSE girls who gave birth in last 3 years was highest in Port Moresby at 91.2%. HIV testing was inconsistently and inadequately offered at ANC across the three cities.

**Conclusions:**

*Kauntim mi tu* provides much-needed insight into the SRH experiences of FSW and CSE girls in PNG, where their use of moderately or highly effective contraceptive methods is low. We hope to shed light on the complicated reality they face due to illegality of sex work and multitude of complex healthcare experiences.

## Background

Female sex workers (FSW), including girls aged < 18 years who are commercially sexually exploited (CSE),^1^ are highly vulnerable and at-risk of HIV and other sexually transmitted infections (STI). They are also at risk of other adverse sexual and reproductive health (SRH) outcomes, including unintended pregnancies, induced abortions, and low levels of antenatal care (ANC) [1, 2]. Globally, the HIV prevalence among FSW and CSE girls is estimated to be 13.5 times greater compared to other women and girls [3]. There is also a high prevalence of bacterial STIs among these vulnerable women and girls [4]. Due to perceived fears or actual experiences with stigmatization and discrimination, these women and girls often do not disclose their engagement in transactional sex and other risky practices to healthcare providers when seeking access to SRH services, severely limiting the ability to be provided with tailored services [5–7]. Furthermore, in many contexts, healthcare providers have insufficient training or guidance on how to assess the SRH needs (and risks) of FSW and CSE girls, which are often more complex than those of women and girls more generally [2, 8]. Therefore, it is paramount that we continue refining our understanding of the SRH needs of FSW and CSE girls before, during, and after pregnancy. Information and lessons learned should then be used to adapt training and guidance materials for healthcare providers caring for these women and girls in their communities to ensure they receive high quality healthcare that is tailored to their needs.

While there are many areas of sexual and reproductive health concern among FSW and CSE girls, one of the least studied aspects in this population is their use of moderately or highly effective contraception, including hormonal based methods. Across Sub-Saharan, Western, and Eastern Africa, these women and girls are reported to experience high rates of pregnancies, both planned and unplanned [8, 9]. Unplanned pregnancies, in particular, have been influenced by a larger number of partners, inconsistent use of condoms or condom negotiation by clients, and sexual violence [2, 8, 9]. In settings where access to safe abortion is criminalized or severely restricted, unplanned pregnancies increase the reproductive and maternal vulnerability of FSW and CSE girls, leading some to resort to unsafe abortions [2, 10].

While some research into the barriers and enablers of HIV and SRH care experienced by FSW and CSE girls has been conducted across the world, relatively little research has sought to understand the experiences of these women and girls in Papua New Guinea (PNG). PNG has the largest HIV epidemic in the Pacific region [11], concentrated among key populations, particularly FSW and CSE girls [12], but also men who have sex with men and transgender women [13]. FSW and CSE girls in PNG have the highest national HIV prevalence in Asia and the Pacific [14], with recent biobehavioral surveys showing HIV prevalence estimates in the largest three cities – Port Moresby, Lae, and Mt. Hagen – are 15.2, 11.9 and 19.6%, respectively [15]. In PNG these women and girls face numerous challenges, including stigma, harassment, physical and sexual violence, and the illegality of sex work, all of which can be barriers to HIV and SRH services [15]. Their experiences are further complicated by ongoing health system challenges in PNG, such as a poorly supported work force and inadequate program coordination [16]. Several studies in PNG have explored the use of condoms among these women and girls examining barriers and enablers to safer sex practices, assessment of HIV and STI knowledge [17, 18], and among women and girls in the general population, exploration of risk factors for low family planning uptake [19]. However, there is an overall knowledge gap for the reproductive health experiences of FSW and CSE girls, including ANC and the use of moderately or highly effective contraceptive methods, which excludes condoms [20]. In this paper we describe the sexual and reproductive health history of FSW and CSE girls and factors associated with their use of moderately or highly effective contraceptive methods, which excludes condoms, in three settings in PNG.

## Methods

### Study criteria

From June 2016 to December 2017 we conducted *Kauntim mi tu* (“Count me too”), a respondent-driven sampling (RDS) biobehavioral survey, in three cities in PNG: Port Moresby (June–October 2016); Lae (January–June 2017); and Mt. Hagen (August–December 2017). RDS builds from snowball sampling and allows us to compensate for the non-random nature of recruitment by the ability to produce sampling weights from the data collected and approximate a random sample [14–16]. Eligibility criteria included: 1) assigned female sex at birth; 2) aged ≥12 years; 3) have sold or exchanged vaginal sex in the past 6 months; 4) could speak English or Tok Pisin; and 5) had a valid RDS study coupon at the time of the study. The low age criterion of ≥12 years was included because of how early some girls in PNG engage in transactional sex and the need for healthcare provider training materials to be adapted to include proper response to sexual exploitation for this particularly vulnerable group of women and girls [18]. Additionally, in PNG, HIV testing is legal without the consent of a guardian or parent from the age of 12 while the legal age to receive family planning without parental consent is 16 years.

### Patient and public involvement

This study was made possible through the extensive collaboration and consultations with Papua New Guinean female sex workers and Friends Frangipani, a local civil society organization representing sex workers, at all phases of the biobehavioral surveys. Community members were involved in formative assessments before study initiation and assisted with activities such as service referrals during data collection. After data collection, findings were reported to members of Friends Frangipani in private meetings who then offered site-specific recommendations that were included in site reports for all three cities [21].

### Data collection

Detailed study methods have been described elsewhere [12, 15, 22, 23]. Briefly, recruitment began with four seeds in Port Moresby, four in Lae, and five in Mt. Hagen. Twelve additional seeds were added in Port Moresby, 15 in Lae, and 4 in Mt. Hagen to facilitate recruitment. Seeds were purposely selected to create diversity with respect to: age, sexual/gender identity, place of residence, region of origin, marital status, and affiliation with a non-governmental or community-based organization. The longest recruitment chain in Port Moresby, Lae, and Mt. Hagen had 26, 19, and 10 recruitment waves, respectively. Consenting participants were interviewed by trained staff members on topics, including their socio-demographics, sexual history, sex work characteristics, reproductive health, pregnancy history, previous and current use of moderately or highly effective contraceptive methods (including pill, injection/depo, implant, IUD, sterilization, patch, or vaginal ring) among those not trying to get pregnant, and HIV testing history. All moderately or highly effective contraceptive methods were hormonal, except for sterilization and non-hormonal IUDs. Condoms were not considered as a moderately or highly effective contraceptive method [24]. Questions related to ANC attendance and testing of HIV and syphilis during their last pregnancy were limited to women whose last live birth was less than or equal to 3 years ago. Currently pregnant FSW and CSE girls were excluded to ensure equal opportunity for ANC attendance and HIV/syphilis testing. Except for HIV status, which was determined using biological testing, all other results are self-reported.

The questionnaire was administered in the language of the participants’ choice (English or Tok Pisin) at their second visit to the survey site and took approximately 1.5 hours to complete. In all locations, participants received 45 PNG kina (about US$14) for their first visit to the survey site and 10 PNG kina (about US$3) per successful recruit plus 5 PNG kina (about US$1.50) for transportation at their second visit.

### HIV testing, treatment, and referral

Following the survey, consenting participants received HIV counseling and testing according to the PNG national algorithm of Determine HIV-1/2 (Alere, Hannover, Germany) with confirmation by Stat-Pak HIV-1/2 (Chembio, New York, NY USA). Individuals testing HIV-positive were also provided testing for CD4 T-cell count using PIMA and HIV viral load using the GeneXpert HIV-1 viral load assay (Cepheid, Sunnyvale, CA). Further details on biological testing were previously published [12].

All participants testing HIV-positive were actively linked to HIV treatment services by a peer navigator. Study staff were trained to identify sexually exploited girls younger than 18 years and refer them to partner organizations experienced in providing psychosocial and protective services to this population.

### Data analysis

Data were analyzed using Respondent-Driven Sampling Analyst version 0.62 (RDS-A, Los Angeles, CA USA) and SAS version 9.3 (Carey, NC USA). Gile’s Successive Sampling Estimator was used in RDS-A. Weighted chi-square tests were calculated to determine if differences in descriptive statistics and relationships between two categorical variables were statistically significant (*P* < 0.05). RDS weights were used in calculations. Odds ratios (OR) and 95% confidence intervals (CI) were calculated and a *P* < 0.1 was the threshold for inclusion in multivariate analysis for each city. Final multivariable models retained variables with a *P* < 0.05 and variables of interest based on adjusted odds ratios that nearly excluded a value of 1.

## Results

We enrolled 2091 FSW and CSE girls who met the study criteria: Port Moresby, 673; Lae, 709; and Mt. Hagen, 709. We successfully reached RDS equilibrium and convergence for our covariates of interest. As seen in Table 1, the median age of these women and girls was significantly different, ranging from 25 to 27 in all three cities. The proportion of CSE girls in our study sites was: Port Moresby (6.6%), Lae (4.6%), and Mt. Hagen (4.2%). The majority of FSW and CSE girls (81.6–86.5%) had only primary or no formal education. Most (68.6–74.5%) were divorced, separated, or widowed and many reported dependent children (37.3–52.0%). Some (14.4–25.0%) had been away from their homes for more than 1 month in the last 6 months.

**Table 1:** Characteristics of female sex workers from a biobehavioral survey *Kauntim mi tu* in Port Moresby, Lae, and Mt. Hagen in Papua New Guinea between 2016 and 2017

	**Port Moresby**			**Lae**			**Mt. Hagen**			
	Valid	Sample proportion	Population proportion^a^	Valid	Sample proportion	Population proportion^a^	Valid	Sample proportion	Population proportion^a^	p-value
		%	% (95% CI)		%	% (95% CI)		%	% (95% CI)	
**Age, years**	633			609			554			<0.001
12-19	77	12.2	14.4 (10.9–18.0 )	73	12.0	11.9 (8.9–15.0)	65	11.7	13.1 (9.8–16.4)	
20-24	173	27.3	28.9 (24.4–33.3)	166	27.3	26.7 (22.4–30.9)	173	31.2	32.4 (27.9–36.9)	
25-29	119	18.8	17.5 (13.9–21.1)	127	20.9	20.2 (16.4–24.0)	135	24.4	23.0 (19.1–26.9)	
30-34	94	14.8	15.0 (11.5–18.5)	105	17.2	19.5 (15.6–23.4)	83	15.0	15.1 (11.7–18.6)	
35 or older	170	26.9	24.2 (20.1–28.2)	138	22.7	21.7 (17.8–25.6)	98	17.7	16.4 (13.0–19.8)	
Median (IQR)	26 (21-34)			27 (22-34)			25 (21-32)			
**Commercially, sexually exploited, ages 12-17**	633			609			554			<0.001
Yes	34	5.4	6.6 (4.0-9.1)	25	4.1	4.6 (2.5-6.7)	20	3.6	4.2 (2.2-6.3)	
**Education**	672			708			709			<0.001
No formal education	163	24.3	23.6 (19.6–27.5)	256	36.2	35.6 (31.4–39.9)	284	40.1	39.9 (35.8–44.0)	
Primary	391	58.2	61.5 (57.0–66.1)	349	49.3	50.9 (46.5–55.3)	295	41.6	41.7 (37.5–45.8)	
High school or other	118	17.6	14.9 (11.6–18.1)	103	14.5	13.5 (10.5–16.4)	130	18.3	18.4 (15.1–21.7)	
**Marital status**	673			708			709			<0.001
Never married	115	17.1	18.2 (14.4–21.9)	137	19.4	19.5 (16.0–23.0)	142	20.0	21.5 (18.0–25.1)	
Married	93	13.8	13.2 (10.1–16.4)	75	10.6	10.5 (7.8–13.2)	26	3.7	4.0 (2.2–5.7)	
Divorced, separated, or widowed	465	69.1	68.6 (64.2–73.0)	496	70.1	70.0 (65.9–74.0)	541	76.3	74.5 (70.7–78.3)	
**Number of dependent children living with FSW **	672			707			709			<0.001
None	321	47.8	48.0 (43.2-52.7)	454	64.2	62.7 (58.4-67.0)	420	59.2	58.8 (54.7-63.0)	
One or more	351	52.2	52.0 (47.3-56.8)	253	35.8	37.3 (33.0-41.6)	289	40.8	41.2 (37.0-45.3)	
**Away from home for >1 month at a time in last 6 months**	650			667			594			<0.001
Yes	91	14.0	14.4 (11.0–17.8)	164	24.6	25.0 (21.0–29.0)	151	25.4	23.7 (20.0–27.5)	
**Sex work as main source of income**	671			701			686			<0.001
Yes	467	69.6	66.5 (61.9–71.1)	458	65.3	62.8 (58.4–67.1)	535	78.0	76.8 (73.1–80.4)	
**Years selling sex**	671			688			638			<0.001
< 1 year	61	9.1	9.4 (6.7–12.2)	42	6.1	6.5 (4.3–8.8)	34	5.3	6.2 (4.0–8.5)	
1-2 years	189	28.2	30.4 (26.0–34.9)	154	22.4	23.7 (19.8–27.6)	158	24.8	26.0 (22.1–29.9)	
3-4 years	134	20.0	21.5 (17.5–25.4)	165	24.0	26.2 (22.1–30.2)	166	26.0	25.8 (21.9–29.7)	
5-9 years	150	22.4	20.1 (16.4–23.8)	194	28.2	28.1 (24.1–32.1)	198	31.0	30.6 (26.5–34.6)	
10 or more years	137	20.4	18.6 (15.0–22.2)	133	19.3	15.5 (12.5–18.4)	82	12.9	11.4 (8.7–14.1)	
**Had a casual or main male partner in last 6 months**	672			708			709			<0.001
Yes	425	63.2	63.4 (58.8-68.0)	378	53.4	55.5 (51.1-59.9)	324	45.7	48.3 (44.0-52.5)	
**Ever tested for HIV**	672			706			708			<0.001
Yes	479	71.3	68.2 (63.7–72.7)	418	59.2	56.1 (51.6–60.5)	439	62.0	60.0 (55.8–64.1)	
**HIV status**	663			704			709			<0.001
Positive	94	14.2	15.2 (11.7–18.8)	83	11.8	11.9 (9.0–14.8)	133	18.8	19.6 (16.1–23.0)	

^a^Estimates are based on RDS weights

The majority (62.8–76.8%) reported sex work as their main source of income. Most of these women and girls had been engaged in sex work for less than 10 years, with between 11.4–18.6% selling sex for 10 or more years. Most FSW and CSE girls (48.3–63.4%) had a casual or main male partner in the last 6 months. Between 56.1–68.2% reported having ever tested for HIV. The estimated HIV prevalence rate varied from 11.9% in Lae to 19.6% in Mt. Hagen (Table 1).

Up to three quarters of all FSW and CSE girls reported one or more pregnancies (64.4–74.1%) and up to one-third (24.0–36.7%) had given birth to a live infant in the last 3 years. A similar proportion (29.9–33.7%) reported trying to become pregnant. Current use of moderately or highly effective contraceptive methods, which excludes condoms, was reported by 26.5–37.7% of these women and girls. Among FSW and CSE girls who have ever been pregnant around one-fifth (18.1–20.5%) tried to induce an abortion (Table 2).

**Table 2:** Reproductive health history of female sex workers from a biobehavioral survey *Kauntim mi tu* in Port Moresby, Lae, and Mt. Hagen in Papua New Guinea between 2016 and 2017

	**Port Moresby**			**Lae**			**Mt. Hagen**			
	Valid	Sample proportion	Population proportion^c^	Valid	Sample proportion	Population proportion^c^	Valid	Sample proportion	Population proportion^c^	p-value
		%	% (95% CI)		%	% (95% CI)		%	% (95% CI)	
**Live birth ≤ 3 years ago**	503			454			464			<0.001
Yes	168	33.4	36.7 (31.3-42.1)	110	24.2	24.0 (19.3-28.7)	122	26.3	26.8 (22.2-31.4)	
**Ever sold sex while pregnant**	298			236			229			0.851
Yes	102	34.2	36.3 (29.4-43.2)	86	36.4	36.2 (28.8-43.6)	89	38.9	37.5 (30.3-44.6)	
**Total number of pregnancies**	672			708			709			<0.001
None	165	24.6	25.9 (21.7-30.2)	250	35.3	35.6 (31.3-39.8)	234	33.0	33.8 (29.7-37.8)	
1 pregnancy	193	28.7	29.3 (25.0-33.6)	205	29.0	26.6 (22.8-30.4)	199	28.1	28.6 (24.8-32.5)	
2-3 pregnancies	215	32.0	29.7 (25.4-33.9)	166	23.4	23.9 (20.1-27.7)	210	29.6	29.5 (25.6-33.3)	
4 or more pregnancies	99	14.7	15.1 (11.7-18.5)	87	12.3	13.9 (10.7-17.2)	66	9.3	8.1 (6.0-10.3)	
**Time since most recent pregnancy **	507			456			476			<0.001
Currently pregnant	9	1.8	1.5 (0.4-2.6)	3	0.7	0.8 (0.0-2.0)	6	1.3		
Within the last 12 months	17	3.4	4.2 (1.9-6.6)	21	4.6	4.8 (2.5-7.2)	26	5.5		
Between 12 months and 3 years ago	155	30.6	32.6 (27.4-37.8)	98	21.5	21.2 (16.7-25.7)	111	23.3		
Longer than 3 years ago	326	64.3	61.7 (56.3-67.0)	334	73.2	73.1 (68.2-78.0)	333	69.9		
**Trying to become pregnant**	663			694			669			<0.001
Yes	190	28.7	29.9 (25.5-34.3)	246	35.4	33.7 (29.5-37.9)	239	35.7		
^**a**^ **Using moderately or highly effective contraceptive methods**	466			446			430			<0.001
Yes	171	36.7	37.7 (32.2-43.2)	131	29.4	30.9 (25.6-36.1)	121	28.1	26.5 (21.9-31.2)	
**Among FSW who have been pregnant, ever tried to induce an abortion**	507			458			481			0.0862
Yes	95	18.7	20.5 (15.9-25.0)	83	18.1	18.6 (14.3-22.8)	92	19.1	18.1 (14.2-22.0)	
^**b**^ **ANC attendance during last pregnancy**	168			110			122			<0.001
Yes	156	92.9	91.2 (85.6-96.9)	94	85.5	86.7 (79.5-94.0)	103	84.4	85.7 (79.1-92.4)	
^**b**^ **Offered an HIV test at an ANC visit during the last pregnancy**	154			93			100			<0.001
Yes	115	74.7	72.4 (63.2-81.5)	73	78.5	74.1 (62.8-85.4)	89	89.0	88.2 (80.4-95.9)	
^**b**^ **Tested for HIV during the last pregnancy**	114			72			89			<0.001
Yes	111	97.4	98.3 (95.8-100.0)	72	100.0	100.0 (98.4-100.0)	89	100	100.0 (98.3-100.0)	
^**b**^ **Trimester in which last tested for HIV**	110			72			89			<0.001
First trimester	8	7.3	5.0 (1.0-9.0)	10	13.9	20.2 (7.3-33.1)	13	14.6	10.6 (4.5-16.6)	
Second trimester	62	56.4	60.5 (49.1-72.0)	45	62.5	62.6 (48.8-76.4)	53	59.6	61.4 (49.8-73.1)	
Third trimester	40	36.4	34.5 (23.3-45.7)	17	23.6	17.2 (8.4-26.0)	23	25.8	28.0 (16.9-39.1)	
^**b**^ **Offered a syphilis test during last pregnancy**	146			91			98			<0.001
Yes	58	39.7	42.9 (32.7-53.1)	26	28.6	28.0 (16.9-39.2)	31	31.6	30.1 (19.6-40.6)	
^**b**^ **Tested for syphilis during last pregnancy**	58			26			31			0.1504
Yes	49	84.5	86.8 (76.3-97.3)	23	88.5	87.6 (72.4-100.0)	25	80.7	79.7 (62.4-97.0)	
^**b**^ **Tested positive for syphilis during last pregnancy**	47			23			24			0.1230
Yes	9	19.2	18.2 (5.1-31.3)	4	17.4	19.9 (0.0-42.9)	2	8.3	4.2 (0.0-10.5)	

^a^Moderately or highly effective contraceptive methods include pill, injection/depo, implant, IUD, sterilized, patch, or vaginal ring

^b^Limited to women whose last live birth was less than or equal to 3 years ago, excluding current pregnancy

^c^Estimates are based on RDS weights

Among FSW and CSE girls who had given birth within the past 3 years, 85.7–91.2% have attended for antenatal care during their last pregnancy. While all FSW and CSE girls were expected to be offered HIV testing, we found 72.4–88.2% were offered an HIV test, and almost all these (98.3–100.0%) tested for HIV. Unlike with HIV, 28.0–42.9% of these women and girls were offered a syphilis test during their last pregnancy. Among these, the majority were tested for syphilis (79.7–87.6%), and 4.2–19.9% tested positive for syphilis (Table 2).

Among FSW and CSE girls in Port Moresby, factors associated with the use of a moderately or highly effective contraceptive method included ages 20–24 (aOR, 2.8; 95% CI: 1.3–6.3), being married (aOR, 4.1; 95% CI: 1.1–14.5), being divorced, separated, or widowed (aOR, 5.8; 95% CI: 1.9–18.2), having one or more depending children living with the FSW or CSE girl (aOR, 1.9; 95% CI: 1.0–3.4), being away from home for more than 1 month in the last 6 months (aOR, 2.6; 95% CI: 1.1–6.2) and having tested negative for HIV (aOR, 2.9; 95% CI: 1.2–7.4) (Table 3). Several factors were associated with the use of a moderately or highly effective contraceptive method in bivariate analysis among FSW and CSE girls in Lae, including having had a casual or main male partner in the last 6 months (OR, 2.1; 95% CI: 1.3–3.5) and having ever tested for HIV (OR, 2.4; 95% CI: 1.4–4.1). However, after adjusting for covariates, no factors were statistically significantly associated with the use of moderately or highly effective contraceptive methods. In Mt. Hagen, several factors were associated with the use of a moderately or highly effective contraceptive method in bivariate analysis, including certain ages and ever having tried to induce an abortion (OR, 1.9; 95% CI: 1.0–3.7). However, no factors were statistically significantly associated with the use of a moderately or highly effective contraceptive method after adjusting for covariates.

**Table 3:** Multivariable analysis of factors associated with the use of moderately or highly effective contraceptive methods among female sex workers from a biobehavioral survey *Kauntim mi tu* in Port Moresby, Lae, and Mt. Hagen in Papua New Guinea between 2016 and 2017

	**Port Moresby**					**Lae**					**Mt. Hagen**				
	**n**	**Bivariate OR** **(95% CI)** ^a^	***p*** **-value**	**Multivariate aOR ** **(95% CI)** ^a^	***p*** **-value**	**n**	**Bivariate OR** **(95% CI)** ^a^	***p*** **-value**	**Multivariate aOR ** **(95% CI)** ^a^	***p*** **-value**	**n**	**Bivariate OR** **(95% CI)** ^a^	***p*** **-value**	**Multivariate aOR ** **(95% CI)** ^a^	***p*** **-value**
**Age, years**	437		0.038		0.031	389		<0.001		0.126	331		0.002		0.055
12-19		0.4 (0.2-1.1)		2.9 (0.5-17.2)			0.6 (0.2-1.8)		2.4 (0.3-21.6)			3.8 (1.1-12.5)		5.2 (0.7-39.3)	
20-24		1.8 (1.0-3.5)		2.8 (1.3-6.3)			3.1 (1.4-6.9)		4.0 (1.4-11.7)			4.6 (1.7-12.2)		4.0 (1.3-12.5)	
25-29		1.1 (0.5-2.2)		0.8 (0.4-1.8)			3.9 (1.7-8.9)		2.8 (1.0-7.4)			7.2 (2.7-19.1)		7.0 (2.4-20.7)	
30-34		1.5 (0.7-3.3)		1.4 (0.6-3.1)			3.5 (1.5-7.9)		3.1 (1.2-8.3)			6.5 (2.3-17.9)		5.3 (1.7-16.9)	
35 or older		Ref		Ref			Ref		Ref			Ref		Ref	
**Marital status**	466		<0.001		0.011	446		0.017		0.392	**-- **		**-- **		**--**
Never married		Ref		Ref			Ref		Ref			**--**		**--**	
Married		5.6 (2.0-15.4)		4.1 (1.1-14.5)			3.0 (1.0-9.4)		0.5 (0.1-2.5)			**--**		**--**	
Divorced, separated, or widowed		6.4 (2.7-14.9)		5.8 (1.9-18.2)			3.3 (1.5-7.4)		1.0 (0.3-2.8)		**-- **		**-- **		**--**
**Number of dependent children living with FSW **	465		<0.001		0.037	446		<0.001		0.209	430		0.005		0.469
None		Ref		Ref			Ref		Ref			Ref		Ref	
One or more		2.5 (1.5-4.0)		1.9 (1.0-3.4)			3.1 (1.9-5.2)		1.5 (0.8-2.8)			2.0 (1.2-3.3)		1.3 (0.6-2.6)	
**Away from home for >1 month at a time, last 6 months**	450		0.011		0.037	**-- **		**-- **		**--**	**-- **		**-- **		**--**
Yes		2.4 (1.2-4.8)		2.6 (1.1-6.2)			**--**		**--**			**--**		**--**	
No		Ref		Ref			**--**		**--**			**--**		**--**	
**Sex work as main source of income**	**-- **		**-- **		**--**	444		0.019		0.300	-		-		-
Yes		**--**		**--**			Ref		Ref			-		-	
No		**--**		**--**			1.8 (1.1-3.0)		1.4 (0.7-2.7)			-		-	
**Years selling sex**	**-- **		**-- **		**--**	438		0.002		0.120	384		0.024		0.287
< 1 year		**--**		**--**			6.1 (2.1-17.9)		5.2 (1.0-26.3)			2.1 (0.6-7.6)		0.4 (0.1-2.7)	
1-2 years		**--**		**--**			4.1 (1.9-8.9)		2.2 (0.8-6.3)			3.4 (1.2-9.4)		1.6 (0.4-6.0)	
3-4 years		**--**		**--**			1.9 (0.9-4.2)		1.0 (0.3-2.6)			5.2 (1.9-14.3)		2.1 (0.6-7.4)	
-9 years		**--**		**--**			2.4 (1.1-5.3)		1.6 (0.6-4.0)			3.4 (1.2-9.1)		1.3 (0.4-4.6)	
10 or more years		**--**		**--**			Ref		Ref			Ref		Ref	
**Had a casual or main male partner in last 6 months**	**-- **		**-- **		**--**	446		0.003		0.076	**-- **		**-- **		**--**
Yes		**--**		**--**			2.1 (1.3-3.5)		1.8 (0.9-3.6)			**--**		**--**	
No		**--**		**--**			Ref		Ref			**--**		**--**	
**Ever tested for HIV**	466		0.097		0.338	446		0.001		0.072	429		0.001		0.143
Yes		1.6 (0.9-2.6)		0.7 (0.4-1.4)			2.4 (1.4-4.1)		1.9 (0.9-3.8)			2.4 (1.4-4.1)		2.0 (0.8-5.1)	
No		Ref		Ref			Ref		Ref			Ref		Ref	
**HIV status**	459		0.008		0.023	**-- **		**-- **		**--**	**-- **		**-- **		**--**
Positive		Ref		Ref			**--**		**--**			**--**		**--**	
Negative		2.9 (1.3-6.2)		2.9 (1.2-7.4)			**--**		**--**			**--**		**--**	
**Live birth ≤ 3 years ago**	387		0.024		0.225	334		0.017		0.503	316		0.030		0.427
Yes		1.8 (1.1-3.2)		1.5 (0.8-3.1)			2.1 (1.1-3.8)		1.3 (0.6-2.7)			1.9 (1.1-3.4)		1.4 (0.6-3.0)	
No		Ref		Ref			Ref		Ref			Ref		Ref	
**Among FSW who have been pregnant, ever tried to induce an abortion**	**-- **		**-- **		**--**	**-- **		**-- **		**--**	324		0.043		
Yes		**--**		**--**			**--**		**--**			1.9 (1.0-3.7)		2.2 (1.0-5.0)	0.056
No		**--**		**--**			**--**		**--**			Ref		Ref	

*Abbreviations*: *OR* odds ratio, *CI* confidence interval, *aOR* adjusted odds ratio

^a^Estimates are based on RDS weights

## Discussion

To our knowledge, our study provides a recent update to multisite data about sexual and reproductive health of FSW and CSE girls in the Pacific region [25]. A low proportion of FSW and CSE girls used moderately or highly effective contraceptive methods, which excludes condoms, in each city (about one-in-three) whereas two-in-three FSW and CSE girls were not trying to become pregnant and one in five FSW and CSE girls who had ever been pregnant tried to induce an abortion. These findings highlight a strong need for expanded SRH services, particularly access to contraception and are contextualized further by ANC attendance and HIV/syphilis testing by these women and girls.

We identified a few factors that were significantly associated with the use of a moderately or highly effective contraceptive method in Port Moresby, but none were associated in Lae and Mt. Hagen after adjusting for covariates. In Port Moresby, FSW and CSE girls ages 20–24, married or divorced, separated, widowed, and had dependent children all had higher odds of using a moderately or highly effective contraceptive method compared to FSW and CSE girls who were 35 or older, never married, and had no dependent children. These demographic characteristics provide a basis for improved targeting of SRH interventions for sub-groups of FSW and CSE girls. Furthermore, FSW and CSE girls who were away from home for more than a month in the last 6 months had nearly three times higher odds of using a moderately or highly effective contraceptive method compared to peers who were not. Future research should explore possible differences in the experiences of women and girls who travel away from home in all three cities. Likewise, all those who tested negative for HIV had more than three times higher odds of using a moderately or highly effective contraceptive method as compared to those who tested HIV positive. In our analyses, almost all the contraceptive methods considered to be moderately or highly effective (six of seven total) were hormonal methods, though some may have been using non-hormonal IUDs. Data from other settings indicate that HIV-positive FSW and CSE girls are less likely to use hormonal contraceptive methods than non-hormonal contraceptive methods [26, 27], a finding reinforced in this study (*p* < 0.0001, data not shown). Based on the multivariate model adjusted odds ratios nearly excluding a value of 1 in Lae, FSW and CSE girls who had a casual or main male partner in the last 6 months had nearly two times higher odds of using a moderately or highly effective contraceptive method compared to women who did not have such partner(s). While no such direct evidence is available, one possibility is that these women and girls wanted to avoid pregnancy due to fear of violence from any of their partners, since physical and sexual violence against FSW and CSE girls in PNG has been previously reported [12]. Secondly, it is plausible based on adjusted odds ratios nearly excluding a value of 1 that FSW and CSE girls who had ever tested for HIV also had nearly two times higher odds of using a moderately or highly effective contraceptive method possibly thanks to accessing health services in general. Without direct evidence, this may be interpreted by acknowledging the reproductive agency of these FSW and CSE girls and them also actively engaging in other health seeking behaviors such as HIV testing [28]. Further research is needed to explore these women and girls’ reproductive decision-making and health agency. The health system should consider the beneficial intersection between HIV and SRH services in Lae. In Mt. Hagen, FSW and CSE girls ages 20–24, 25–29, and 30–34 all had higher odds of using a moderately or highly effective contraceptive method compared to peers 35 or older, providing an opportunity for improved targeting of SRH interventions. Moreover, it is plausible based on adjusted odds ratios nearly excluding a value of 1 that FSW and CSE girls who had tried to induce an abortion had over two times higher odds of using a moderately or highly effective contraceptive method compared to those who did not. This suggests that these women and girls who had unwanted pregnancies in the past may be more aware of contraceptive methods and therefore more likely to use these methods in the future, perhaps due to strong outreach by NGOs in this city relative to Port Moresby and Lae. With nearly half of all FSW and CSE girls having two or more pregnancies in their lifetime, coupled with nearly one-in-three giving birth within the last 3 years, these women and girls in PNG have substantial reproductive health needs.

ANC attendance amongst FSW and CSE girls who gave birth in the last 3 years was highest in Port Moresby at 91.2%; compared to Lae and Mt. Hagen (86.7 and 85.7%, respectively). These figures are higher than the national estimate for PNG women and girls in the general population (76.1%) [29], indicating that they have better means of accessing ANC in these three cities. This may be due to outreach and engagement activities carried out by sex work community organizations or them accessing HIV-related services and being further referred to reproductive health services.

Increasing awareness and access to moderately or highly effective contraceptive methods could prevent unwanted pregnancies. While all young girls 16 years and older are legally able to receive family planning without parental consent, in reality it is often restricted to married women and girls. As the women and girls in our study are mostly unmarried (86.8%), we suggest the health system provide equitable access to family planning as well as integration of SRH and HIV services. Much like lowering the age at which a person can get an HIV test has helped increase HIV testing among adolescents, lowering the age at which they can receive family planning can improve the sexual and reproductive health of adolescent females. It is additionally important to recognize that one-in-three FSW and CSE girls reported selling sex while pregnant. Given their increased risk for HIV and other STIs, we suggest that these women and girls receive health services that cater to their profession, such as promotion of pre-exposure and post-exposure prophylaxis, condoms, lubricants, more frequent HIV testing, psychosocial support, and ART support for HIV positive individuals. Moreover, we suggest the healthcare sector increase the safety and wellbeing of FSW and CSE girls by considering both their reproductive health needs and disease prevention needs simultaneously.

Across all three sites, FSW and CSE girls are inconsistently and inadequately offered HIV testing at ANC. This highlights the shortcomings of the broader prevention of mother-to-child transmission program in the country where improvements should be made [30]. Other research reports that clinics are often understaffed relative to their patient volume and staff are not always trained on preventing mother-to-child HIV transmission [16]. Furthermore, among women and girls from our study whose last live birth was less than or equal to 3 years ago and who were tested for HIV, the majority of their tests did not occur in the first trimester. A substantial proportion reporting that their tests were done as late as the third trimester, increasing risk of vertical transmission. Other studies indicate that ANC attendance during the first trimester varies across the region, with an estimate of 50% in Asia, 17.4% in PNG, and as low as 4.3% in Vanuatu and larger proportions of women and girls have their first ANC visit after their first trimester [29, 31–33]. These findings are also crucial since PNG national and provincial data does not disaggregate data of FSW and CSE girls from women and girls in the general population. HIV testing at ANC visits during certain trimesters is likely, then, to reflect the timing of when FSW attend their first ANC visit and explain why they tested so late in their pregnancies. The health system should increase these women’s and girls’ earlier access ANC services and ensure that more women and girls are seeking HIV testing earlier in their pregnancy.

The low proportion of all women and girls in PNG being offered and tested for syphilis during pregnancy is an issue as well. Due to unavailability and reduced laboratory capacity, STI screening and testing in PNG is limited, so PNG relies on syndromic management [5]. Given the high burden of asymptomatic STI infections, many STI infections are missed. The need for STI screening and subsequent treatment for all women is vast while recognizing that FSW and CSE girls are at increased risk of infections due to their engagement in transactional sexual relationships. Prevalence of chlamydia, gonorrhea, and syphilis among FSW and CSE girls in PNG has been reported as high as 31.5, 21.4, and 7.1%, respectively [12, 34]. As previous research has indicated [5], there is a need to integrate the many vertical health services currently available in PNG within a “one-stop-shop” model. Further research is also needed to understand if/when FSW and CSE girls stop selling sex during pregnancy and if/when they resume selling sex after giving birth as well as their SRH and ANC visit experiences. Such information would be crucial to better characterizing the risks of vertical HIV transmission.

This study has limitations. Our study findings are limited by the self-reported nature of the interview data and the cross-sectional nature of the surveys in each of the three sites. Because interviews were conducted face-to-face, our data may feature response bias, which could be mitigated in the future using audio-computer-assisted self-interviews. Our findings, while representative of the network of FSW and CSE girls recruitment accessed, may not be representative of all FSW and CSE girls. Although seeds were selected with a view toward diversity, it is possible that our findings are not representative. To help mitigate this, investigators routinely monitored data during data collection for homophily, bottlenecks, and convergence. Seeds were added as needed in response to homophily and bottlenecks. Furthermore, the study had extensive collaboration with Papua New Guinean FSW and CSE girls and Friends Frangipani, a local CSO to minimize any additional biases. Highest level of education varied significantly across the three cities which may have impacted reporting. Furthermore, investigators learned during data collection that FSW and CSE girls in Lae and Mt. Hagen were less likely to know their specific age. As a result, some FSW and CSE girls were removed from the analyses in Lae and Mt. Hagen. Lastly, as our study was on FSW and CSE girls and did not include women and girls from the general population we are unable to make direct comparisons between the two populations.

## Conclusions

*Kauntim mi tu* showcases the reproductive health experiences of FSW and CSE girls in PNG and hopes to shed light on the multitude of complex experiences that these women and girls face in the healthcare setting. We hope that future research explores the SRH experiences of FSW in greater detail to understand the challenges that these women and girls face in their personal and professional lives. The complicated reality faced by FSW and CSE girls in PNG, especially due to the illegality of sex work, provides a much-needed opportunity for collaboration between public health programs, healthcare providers, civil society organizations supporting FSW and CSE girls, and these women and girls themselves.

## Data Availability

Data are not publicly available due to ethical considerations surrounding work with these vulnerable and key populations in Papua New Guinea, where sex work is illegal and stigmatized. Data can be made available upon reasonable request to the corresponding author.

## Abbreviations

ANC: Antenatal care
CDC: US Centers for Disease Control and Prevention
CI: Confidence intervals
CSE: Commercially sexually exploited
FSW: Female sex workers
OR: Odds ratios
PNG: Papua New Guinea
RDS: Respondent-driven sampling
STI: Sexually transmitted infection
SRH: Sexual and reproductive health

## Footnotes

1 Following international health law, persons aged < 18 years who sell sex in exchange for goods, services, or money are referred to as victims of sexual exploitation.
